# Artificial intelligence in the practice of forensic medicine: a scoping review

**DOI:** 10.1007/s00414-023-03140-9

**Published:** 2023-12-13

**Authors:** Laurent Tournois, Victor Trousset, Didier Hatsch, Tania Delabarde, Bertrand Ludes, Thomas Lefèvre

**Affiliations:** 1 Université Paris Cité, CNRS UMR 8045, 75006 Paris, France; 2 BioSilicium, Riom, France; 3 IRIS Institut de Recherche Interdisciplinaire Sur Les Enjeux Sociaux, UMR8156 CNRS – U997 Inserm – EHESS – Université Sorbonne Paris Nord, Paris, France; 4 Department of Forensic and Social Medicine, AP-HP, Jean Verdier Hospital, Bondy, France; 5 Institut Médico-Légal de Paris, Paris, France

**Keywords:** Artificial intelligence, Forensic medicine, Medicolegal practice, Forensic pathologist, Routine

## Abstract

Forensic medicine is a thriving application field for artificial intelligence (AI). Indeed, AI applications intended to forensic pathologists or forensic physicians have emerged since the last decade. For example, AI models were developed to help estimate the biological age of migrants or human remains. However, the uses of AI applications by forensic pathologists or physicians and their levels of integration in medicolegal practices are not well described yet. Therefore, a scoping review was conducted on PubMed, ScienceDirect, and Scopus databases. This review included articles that mention any AI application used by forensic pathologists or physicians in practice or any AI model applied in one expertise field of the forensic pathologist or physician. Articles in other languages than English or French or dealing mainly with complementary analyses handled by experts who are not forensic pathologists or physicians or with AI to analyze data for research purposes in forensic medicine were excluded from this review. All the relevant information was retrieved in each article from a grid analysis derived and adapted from the TRIPOD checklist. This review included 35 articles and revealed that AI applications are developed in thanatology and in clinical forensic medicine. However, those applications seem to mainly remain in research and development stages. Indeed, the use of AI applications by forensic pathologists or physicians is not actual due to issues discussed in this article. Finally, the integration of AI in daily medicolegal practice involves not only forensic pathologists or physicians but also legal professionals.

**Supplementary Information:**

The online version contains supplementary material available at 10.1007/s00414-023-03140-9.

## Introduction

Since the last decade, artificial intelligence (AI) is developing in almost all industries [1]. Particularly, AI applications have emerged in expertise fields, such as medicine [2], justice, and criminal law [3]. In addition, AI is expected to be developed in recent fields of medicine. For instance, in P5 (predictive, personalized, preventive, participatory, and psycho-cognitive) medicine, AI would support decision-making processes as well as diagnoses and prognoses [4].

Nowadays, AI may be considered as a modeling tool for specific tasks [5]. For example, an AI model may be specifically designed to detect breast cancer from mammograms [6]. In this review, an AI application is considered as a model integrated in a computer program or a part of a computer program that performs a specific task. This model can be built from data such as numerical or categorical variables, images, texts, or rules.

Therefore, one may expect to find AI applications developed for forensic medicine purposes in the literature. Besides, Tournois and Lefèvre gave an overview of the AI applications used by forensic pathologists or physicians in daily practice [7]. In this review, a systematic and reproducible method is provided to establish a state-of-the-art on the daily use of AI by forensic pathologists or physicians. Since scoping reviews are more indicated for providing evidence to inform practice than systematic reviews [8], a scoping review approach is proposed in this article. The objectives are to (i) identify the AI applications used by forensic pathologists or physicians and (ii) map the AI landscape in the expertise fields of forensic medicine by estimating the level of integration or maturity of the identified AI applications.

## Methods

### Protocol and registration

In this scoping review, the protocol was defined and adapted from the Preferred Reporting Items for Systematic Reviews and Meta-analysis Protocols extension for scoping reviews (PRISMA-ScR) [9]. Since scoping reviews are not allowed for registration on PROSPERO, no process of registration was performed. Therefore, no registration number was assigned to this review.

### Information sources and search strategy

The articles were extracted from the PubMed, ScienceDirect, and Scopus databases from inception to September 28, 2022, using search queries described in Table 1.

**Table 1:** Search queries used for each database in order to extract articles for screening

Database	Search query
PubMed (MeSH terms)	(“Artificial Intelligence”[MeSH] OR “Decision Trees”[MeSH] OR “Neural Networks, Computer”[MeSH] OR “Decision Support Techniques”[MeSH]) AND (“Autopsy”[MeSH] OR “Thanatology”[MeSH] OR “Forensic Medicine”[MeSH]) NOT (“Genomics”[MeSH] OR “DNA”[MeSH] OR “RNA” [MeSH] OR “Forensic Genetics”[MeSH] OR “Forensic Toxicology”[MeSH] OR “Forensic Dentistry”[MeSH] OR “Forensic Ballistics”[MeSH] OR “Forensic Entomology”[MeSH] OR “Forensic Psychiatry”[MeSH] OR “Forensic Psychology”[MeSH] OR “Biometric Identification”[MeSH] OR “Forensic Anthropology”[MeSH] OR “Blood Stains”[MeSH] OR “Dermatoglyphics”[MeSH] OR “DNA Fingerprinting”[MeSH] OR “Legal Epidemiology”[MeSH] OR “Lie Detection”[MeSH] OR “Paternity”[MeSH] OR “Research”[MeSH])
PubMed (Text words)	((“forensic medicine”[Text Word] OR “forensic pathologist*”[Text Word] OR “forensic physician*”[Text Word] OR “forensic medical doctor*”[Text Word] OR “medicolegal pathologist*”[Text Word] OR “medicolegal physician*”[Text Word] OR “medicolegal doctor*”[Text Word]) AND (“artificial intelligence”[Text Word] OR “algorithm*”[Text Word] OR “machine learning”[Text Word] OR “deep learning”[Text Word] OR “statistical learning”[Text Word] OR “supervised learning”[Text Word] OR “unsupervised learning”[Text Word] OR “semi-supervised learning”[Text Word] OR “predictive modeling”[Text Word] OR “clustering”[Text Word] OR “dimensionality reduction”[Text Word] OR “ensemble method*”[Text Word] OR “transfer learning”[Text Word] OR “reinforcement learning”[Text Word] OR “feature selection”[Text Word] OR “decision support”[Text Word] OR “neural network*”[Text Word] OR “expert system*”[Text Word] OR “multi-agent system*”[Text Word] OR “multiagent system*”[Text Word] OR “decision tree*”[Text Word] OR “random forest*”[Text Word] OR “gradient boosting”[Text Word] OR “logistic regression*”[Text Word] OR “support vector machine*”[Text Word] OR “Bayesian network*”[Text Word] OR “Naïve Bayes”[Text Word] OR “natural language processing”[Text Word] OR “computer vision”[Text Word] OR “Markov decision*”[Text Word] OR “genetic algorithm*”[Text Word] OR “fuzzy model*”[Text Word] OR “fuzzy logic*”[Text Word] OR heuristic*[Text Word])) NOT (genomic*[Text Word] OR DNA[Text Word] OR RNA[Text Word] OR “digital forensic*”[Text Word] OR genetic*[Text Word] OR toxicolog*[Text Word] OR dentistry[Text Word] OR odontolog*[Text Word] OR ballistic*[Text Word] OR entomolog*[Text Word] OR psychiatr*[Text Word] OR psycho*[Text Word] OR biometr*[Text Word] OR anthropolog*[Text Word] OR “blood stain*”[Text Word] OR dermatoglyphic*[Text Word] OR “DNA fingerprint*”[Text Word] OR epidemiolog*[Text Word] OR “lie detection”[Text Word] OR paternity[Text Word] OR research*[Text Word])
ScienceDirect	(“forensic medicine” OR “forensic pathologist” OR “forensic physician” OR “forensic medical doctor” OR “medicolegal pathologist” OR “medicolegal physician” OR “medicolegal doctor”) AND (“artificial intelligence”) AND (-DNA)
Scopus	( TITLE-ABS-KEY ( ( ( {forensic medicine} OR {forensic pathologist*} OR {forensic physician*} OR {forensic medical doctor*} OR {medicolegal pathologist*} OR {medicolegal physician*} OR {medicolegal doctor*}) AND ( {artificial intelligence} OR {algorithm*} OR {machine learning} OR {deep learning} OR {statistical learning} OR {supervised learning} OR {unsupervised learning} OR {semi-supervised learning} OR {predictive modeling} OR {clustering} OR {dimensionality reduction} OR {ensemble method*} OR {transfer learning} OR {reinforcement learning} OR {feature selection} OR {decision support} OR {neural network*} OR {expert system*} OR {multi-agent system*} OR {multiagent system*} OR {decision tree*} OR {random forest*} OR {gradient boosting} OR {logistic regression*} OR {support vector machine*} OR {Bayesian network*} OR {natural language processing} OR {computer vision} OR {Markov decision} OR {genetic algorithm*} OR {fuzzy model*} OR {fuzzy logic*} OR heuristic*)) AND NOT ( genomic* OR {DNA} OR {RNA} OR {digital forensic*} OR {genetic*} OR {toxicolog*} OR {dentistry} OR {odontolog*} OR {ballistic*} OR {entomolog*} OR {psychiatr*} OR {psycho*} OR {biometr*} OR {anthropolog*} OR {blood stain*} OR {dermatoglyphic*} OR {DNA fingerprint*} OR {epidemiolog*} OR {lie detection} OR {paternity} OR {research*})) AND ( LANGUAGE ( english) OR LANGUAGE ( french)))

### Eligibility criteria

Articles were selected if there was any mention of AI used by forensic pathologists or physicians in practice or if articles described explicitly AI applications in one expertise field of forensic medicine. Those expertise fields include postmortem identification, postmortem interval estimation, the determination of the causes of death, and the clinic examination of living persons in a forensic context. However, articles were excluded if they mainly dealt with complementary analyses handled by experts who are not medical doctors in the fields of forensic toxicology, entomology, dentistry, anthropology, psychology, epidemiology, biometrics, and ballistics. Articles were also excluded from this review if they were published in a different language than English or French or if they mainly talked about the use of AI to analyze data for research purposes in forensic medicine. Only the articles with an available abstract in English or French were retrieved.

### Selection of sources of evidence

The selection of articles was independently and blindly performed by two reviewers (LT, VT), on the basis of titles and abstracts by taking into account the eligibility criteria. A third reviewer (TL) selected the articles that were subject to disagreements between both the previous reviewers. The selected reviews were not included as reports; however, their references were included if they met the eligibility criteria.

### Data charting process and data items

After article selection, the inclusion of articles in the scoping review was determined by a reviewer (LT) through the analysis of the whole text of articles. This analysis was performed with an analysis grid (see Table 2) derived from the TRIPOD checklist [10] and validated by the other two reviewers (TL, VT). It is important to mention that the final user of AI applications is rarely explicit in titles or abstracts. Therefore, articles describing AI applications for which the forensic pathologist or physician was not the final user were excluded from the review.

**Table 2:** Analysis grid used to extract relevant information from reports. aTRL: adapted technology readiness level

Domain	Criterion
Publication type	Original article, communication, conference paper, book, technical note
Publication reliability	Peer-reviewed publication, date of the publication
Data sources	Real or generated data, subject types (humans or animals)
Population/sample study	Representativeness of the population/sample, inclusion, and exclusion criteria
	Size of the population/sample
Input data	Features (input data) used for the model
	Datasets used and distribution of data (balanced vs. unbalanced data)
	Processing of missing data
Outcome	Description of the outcome variable
Model development	Architecture of the model
	How overfitting is handled
Model performance	Metrics used to assess the performance of the model
Model evaluation	Value of the performance metrics
Real application	Is the model used in medicolegal practice? Does it perform better compared to other non-AI methods?
	Is the model applied on a population/sample that is included into the population/sample study?
Maturity of the application	Maturity of the application estimated from the aTRL scale

The level of maturity of AI applications described in the selected articles was then assessed by using an adapted Technology Readiness Level (aTRL) scale. The TRL scale is originally defined by 7 values augmented to 9 values corresponding to the maturity of a technology from the observation of the basic concepts behind that technology to its use in practice with success [11]. However, this version of the TRL scale is not suitable for the assessment of the maturity of AI applications in forensic medicine. First, the levels described in the original scale were specifically designed for aero-spatial applications. Second, the number of levels in this scale is not compatible with the details of information extracted from the selected articles. Therefore, levels of technology maturity must be adapted for forensic medicine applications. That is why this original TRL scale was reduced to 3 values corresponding to the formulation of the AI application (aTRL = 1), the stages of research and development of the AI model (aTRL = 2), and its use in daily practice (aTRL = 3).

### Synthesis of results

The studies were grouped by expertise field of the forensic pathologist or physician, that is to say postmortem identification, the determination of the causes of death, and the estimation of the postmortem interval and clinical forensic medicine. For each expertise field, the number of articles and the highest aTRL were summarized to specifically assess the level of development and integration of AI by expertise field.

## Results

### Selection of the sources of evidence

**Fig. 1:**
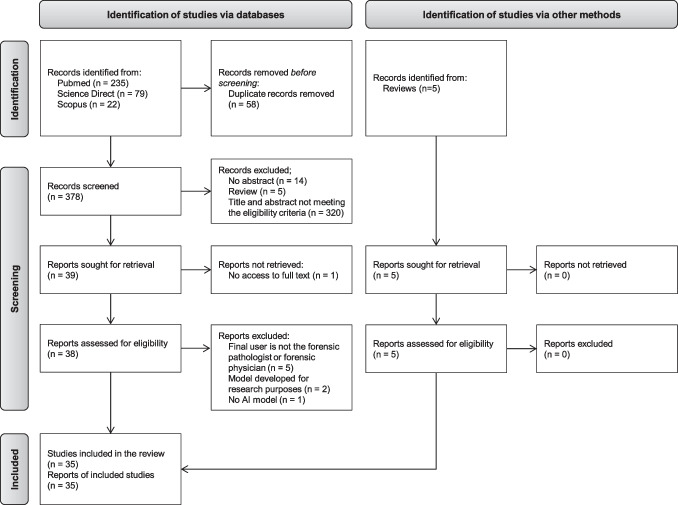
Selection of sources of evidence. Adapted from Page et al. 2021 [12]

The systematic search of the literature results in 436 records. After duplicates removal, 378 records are selected for screening. Based on the titles and the abstracts, 339 are excluded with 39 reports sought for retrieval and eligibility assessment on the full text. Among those reports, 1 is excluded because the full text is not accessible without reader registration. The application of the eligibility criteria to the full text of the 38 remaining reports leads to the exclusion of 8 reports, with 5 reports for which the final user of the AI model is not the forensic pathologist or physician, 2 reports that describe an AI model for research purposes only, and 1 report which does not describe any AI model. From this screening process, 30 articles are eligible in this review. It is worth mentioning that 5 reviews are identified along the screening process. However, the description of the AI applications in those reviews is not detailed enough to assess the performance and the level of maturity as well as applications described from primary sources. Therefore, those reviews are excluded. Nevertheless, the cited references within reviews are analyzed and included if they meet the eligibility criteria. This leads to include 5 reports from reviews. A total of 35 studies are thus included in this review (Fig. 1).

### Characteristics of the sources of evidence

The characteristics of source evidence are described in Table 3. For each included study, the type of article and the purpose of the AI applications are summarized. The 35 studies show that AI applications are developed in thanatology, especially for postmortem identification [13–19], the estimation of the postmortem interval [20–22], and the determination of the causes of death [23–31]. In clinical forensic medicine, AI models are mainly designed for age estimation [15, 32–44] and gender determination [15–17, 45]. One AI model is aimed for the assessment and management of risk of violent reoffending among prisoners [46] and one for bruises dating [47].

**Table 3:** Characteristics of sources of evidence. *CT*, computed tomography; *MRI*, magnetic resonance imaging; *PMCT*, postmortem computed tomography

Reference	Type of article	Purpose of the AI application
Karasik et al. 1999 [32]	Original article	Estimation of the chronological age from roentgenographs of the hand
Karasik et al. 2000 [33]	Original article	Estimation of the chronological age from roentgenographs of the hand in 9 populations
Bocaz-Beneventi et al. 2002 [20]	Original article	Estimation of the postmortem interval using electrolytes measurements in human vitreous humor
Constantinou et al. 2015 [46]	Original article	Risk assessment of violent reoffending among prisoners
Simmons et al. 2016 [13]	Original article	Distinction between human and non-human cranial bone in burnt and unburnt state
Stern et al. 2016 [34]	Conference paper	Estimation of biological age from hand MRI volumes
Yilmaz et al. 2017 [23]	Technical note	Determination of live or stillbirth death
Ebert et al. 2017 [24]	Original article	Detection and localization of hemopericardium from PMCT images
Spampinato et al. 2017 [35]	Original article	Estimation of bone age from X-ray hand images
Stern et al. 2017 [36]	Conference paper	Estimation of chronological age from skeletal and dental MRI volumes and distinction between majority and minority age
Zhang et al. 2018 [37]	Original article	Estimation of skeletal age from costal cartilage maturity ossification stages
Canturk et al. 2018 [21]	Original article	Estimation of the postmortem interval from eye opacity
Heimer et al. 2018 [25]	Original article	Detection of fracture or intact skull
Koterova et al. 2018 [14]	Original article	Age estimation of adult human remains from hip bones
Matoba et al. 2018 [26]	Original article	Estimation of lung weight from PMCT images
Stern et al. 2019 [38]	Original article	Biological and chronological age estimation from MRI volumes of the hand
Andersson et al. 2019 [22]	Original article	Estimation of postmortem interval from partial body scores
Avuclu et al. 2019 [15]	Original article	Determination of age and gender from dental X-ray images
De Back et al. 2019 [39]	Conference paper	Estimation of chronological age from orthopantomograms
Li et al. 2019 [40]	Original article	Estimation of chronological age from pelvic X-ray images
Milosevic et al. 2019 [16]	Conference paper	Determination of gender from orthopantomograms
Turan et al. 2019 [17]	Original article	Determination of gender from bone length measurement of the first and fifth phalanges and metatarsals
Abderrahmane et al. 2020 [41]	Conference paper	Estimation of chronological age from hand photographs
Garland et al. 2020 [27]	Original article	Detection of fatal head injuries
Homma et al. 2020 [28]	Conference paper	Drowning diagnosis from postmortem lung CT images
Peleg et al. 2020 [18]	Original article	Determination of gender from sternum and rib morphology
Pena-Solorzano et al. 2020 [19]	Original article	Localization of femur and detection of orthopedic implants
Tirado et al. 2020 [47]	Technical note	Bruise dating from photographs
Vila-Blanco et al. 2020 [42]	Original article	Estimation of chronological age from orthopantomograms
Mauer et al. 2021 [43]	Original article	Estimation of chronological age from 3D knee MRI images
Ozdemir et al. 2021 [44]	Original article	Estimation of bone age from radiographs
Oura et al. 2021 [29]	Original article	Estimation of the gunshot distance from photographs
Garland et al. 2021 [30]	Original article	Classification of myocardial infarction from histopathology slides
Ibanez et al. 2022 [31]	Original article	Detection of rib fractures from PMCT images
Li et al. 2022 [45]	Original article	Gender determination from pelvic anteroposterior radiographs

### Results of the individual sources of evidence

The results of the individual sources of evidence are summarized in Table 4. For each included study, the aim, the type, the performance, and the maturity level of AI applications are summarized. The detailed results are available in Online Resource 1. The results show that all the included studies remain in research and development stages (aTRL = 2). Moreover, the performance of AI applications may seem too low for a use of developed AI models in daily practice. Indeed, if a low-performance model is considered as a model with performance metrics lower than 90% for classification tasks and greater than 1 year for age estimation error, then 22 AI applications [14, 16, 18–21, 23–28, 33, 36–43, 46] will not be performant enough for a medicolegal usage. Therefore, AI models seem not to be used in daily practice by forensic pathologists and physicians.

**Table 4:** AI performance and integration level for each AI application included in this review. *AUC*, area under the ROC curve; *LR*, likelihood ratio; *MAE*, mean absolute error; *RMSE*, root mean square error; *R*^2^, correlation coefficient

Reference	Aim of the AI application	Type of AI algorithm	AI model performance	aTRL
Karasik et al. 1999 [32]	Age estimation for clinical forensic medicine purposes	Multilinear regression	R^2^ between estimated and ground truth values ranges between 0.818 and 0.901	2
Karasik et al. 2000 [33]	Age estimation for clinical forensic medicine purposes	Logistic regression	*R*^2^ between estimated and ground truth values ranges between 0.671 and 0.901. Standard error estimate ranges between 4.22 and 6.64 years	2
Bocaz-Beneventi et al. 2002 [20]	Estimation of postmortem interval	Artificial neural network	Average residual of the difference between the estimated and experimental values on the validation set is 3.04 h	2
Constantinou et al. 2015 [46]	Assessment of the risk of violent reoffending	Bayesian network	AUC is 0.78	2
Simmons et al. 2016 [13]	Postmortem identification	Decision tree	Accuracy is 1	2
Stern et al. 2016 [34]	Age estimation for clinical forensic medicine purposes	Deep convolutional neural network	Best MAE is 0.36 ± 0.3 years	2
Yilmaz et al. 2017 [23]	Determination of the causes of death	Artificial neural network, logistic regression and radial-basis function network	Specificity is 0.833, sensitivity is 1, *F* score is 0.9091, accuracy is 0.9	2
Ebert et al. 2017 [24]	Determination of the causes of death	Artificial neural network (architecture is not described)	Detection task: average precision, sensitivity and *F* score are respectively 0.85 ± 0.11, 0.77 ± 0.26, 0.77 ± 0.16 Segmentation task: average precision, sensitivity, and *F* score are respectively 0.79 ± 0.05, 0.78 ± 0.05, 0.78 ± 0.0003	2
Spampinato et al. 2017 [35]	Age estimation for clinical forensic medicine purposes	Convolutional neural network with regression network	MAE is 0.79 years	2
Stern et al. 2017 [36]	Age estimation for clinical forensic medicine purposes	Random forest and convolutional neural network (architecture is not described)	Age estimation: MAE is 1.14 ± 0.96 years Majority age distinction: accuracy is 0.913, sensitivity is 0.886, and specificity is 0.932	2
Zhang et al. 2018 [37]	Age estimation for clinical forensic medicine purposes	Linear regression, support vector machine, decision tree, and gradient boosting	MAE is 5.31 years for males and 6.72 years for females	2
Canturk et al. 2018 [21]	Estimation of postmortem interval	Support vector machine and *k*-nearest neighbors	Best accuracy is 0.89	2
Heimer et al. 2018 [25]	Determination of the causes of death	Artificial neural network (architecture is not described)	AUC is 0.965, sensitivity is 0.914, and specificity is 0.875	2
Koterova et al. 2018 [14]	Postmortem identification	Artificial neural network, decision tree, M5 tree, *k*-nearest neighbors, multilinear regression model, and collapsed regression model	MAE is 9.7 years and RMSE is 13.3 years	2
Matoba et al. 2018 [26]	Determination of the causes of death	Multivariate linear regression	*R*^2^ between estimated and real lung weight is 0.89	2
Stern et al. 2019 [38]	Age estimation for clinical forensic medicine purposes	Convolutional neural network	Biological age estimation: best MAE is 0.2 ± 0.42 years Chronological age estimation: best MAE is 0.82 ± 0.65 years Distinction of majority age: AUC is 0.9568	2
Andersson et al. 2019 [22]	Estimation of postmortem interval	Bayesian network (architecture is not described)	LR < 1	2
Avuclu et al. 2019 [15]	Age estimation and determination of gender for clinical forensic medicine purposes and postmortem identification	Multilayer perceptron	Age estimation: difference between predicted and true age ranges from 0 to 6 years Gender determination: success rate between 1.5 and 100% depending on the method used to preprocess teeth images	2
De Back et al. 2019 [39]	Age estimation for clinical forensic medicine purposes	Bayesian convolutional neural network	Overall MAE is 21 months	2
Li et al. 2019 [40]	Age estimation for clinical forensic medicine purposes	Convolutional neural network	MAE is 0.89 years and RMSE is 1.21 years	2
Milosevic et al. 2019 [16]	Determination of gender for clinical forensic medicine purposes and postmortem identification	Convolutional neural network	Accuracy is 0.9687 ± 0.0096	2
Turan et al., 2019 [17]	Determination of gender for clinical forensic medicine purposes and postmortem identification	Multilayer perceptron	Accuracy if 0.965, sensibility is 0.956, specificity is 0.973, and Matthews correlation coefficient is 0.929	2
Abderrahmane et al. 2020 [41]	Age estimation for clinical forensic medicine purposes	Convolutional neural network combined with gated recurrent units	MAE is 1.9266 years	2
Garland et al. 2020 [27]	Determination of the causes of death	Convolutional neural network (architecture is not described)	Accuracy is 0.7	2
Homma et al. 2020 [28]	Determination of the causes of death	Convolutional neural network	AUC is 0.879	2
Peleg et al. 2020 [18]	Postmortem identification	Multivariate linear regression	Success rate ranges from 0.667 to 0.89	2
Pena-Solorzano et al. 2020 [19]	Postmortem identification	Residual networks, hybrid convolutional auto-encoder and *K*-nearest neighbors	Localization of femur: MAE, Jaccard similarity coefficient, and Dice score respectively range between 0 and 13.1 mm, 0.91 and 1, ranges between 0.93 and 1 Detection of implants: Accuracy, precision, recall, and *F*-score respectively range between 0.97 and 1, 0.91 and 0.99, 0.65 and 1, and 0.76 and 0.98	2
Tirado et al. 2020 [47]	Bruise dating	Convolutional neural network	Sensitivity and precision are 0.97, and specificity is 0.995	2
Vila-Blanco et al. 2020 [42]	Age estimation for clinical forensic medicine purposes	Convolutional neural network	*R*^2^ is 0.9, accuracy is 0.854, sensitivity is 0.878, specificity is 0.823, and AUC is 0.925	2
Mauer et al. 2021 [43]	Age estimation for clinical forensic medicine purposes	Convolutional neural network + tree-based machine learning algorithm	MAE is 0.71 ± 0.55 years for the coronal and 0.81 ± 0.62 years for the sagittal dataset Best accuracy, sensitivity, specificity, and AUC are respectively 0.875, 0.884, 0.886, and 0.943 for the sagittal dataset and 0.857, 0.864, 0.846, and 0.908 for the coronal dataset	2
Ozdemir et al. 2021 [44]	Age estimation for clinical forensic medicine purposes	Convolutional neural network	Kütahya Child Radiology Dataset: best MAE, RMSE, and *R*^2^ are 4.3, 5.76, and 0.99 respectively. Radiological Society of North America dataset: best MAE, RMSE, and *R*^2^ are 5.75, 7.42, and 0.96 respectively. The units of the performance metrics are not clear (years or months)	2
Oura et al. 2021 [29]	Determination of the causes of death	Multilayer perceptron	Testing accuracy and F1 range from 0.94 to 1, recall ranges from 0.89 to 1, precision from 0.92 to 1, and AUC from 0.99 to 1. Averaged test accuracy is 0.98	2
Garland et al. 2021 [30]	Determination of the causes of death	Convolutional neural network	Accuracy and F1 scores are equal to 1	2
Ibanez et al. 2022 [31]	Determination of the causes of death	Convolutional neural network	Recall, precision, and F1 score are respectively 0.93 ± 0.05, 0.89 ± 0.03, and 0.91 ± 0.04	2
Li et al. 2022 [45]	Gender determination for clinical forensic medicine purposes	Convolutional neural network	Average accuracy is 0.946 in Chinese Han population and 0.829 in White population	2

### Synthesis of results

In summary, 35 AI applications are identified for a use by forensic pathologists or physicians in thanatology and forensic clinical medicine respectively (Table 5). In thanatology, 19 AI models may help forensic pathologists identify deceased individuals, estimate the postmortem interval, or determine of the causes of death. In forensic clinical medicine, 19 AI models may help forensic physicians estimate the age of young individuals, date bruises in physical assault contexts, and assess the risk of violent reoffending of prisoners. However, no AI application identified in this review seems to be currently used in daily medicolegal practice by forensic pathologists or physicians (aTRL = 2).

**Table 5:** Synthesis of results by expertise field. *aTRL*, adapted technology readiness level

Expertise field	References	Number of articles	Highest aTRL
Postmortem identification	[13–19]	7	2
Postmortem interval estimation	[20–22]	3	2
Determination of the causes of death	[23–31]	9	2
Age estimation in clinical forensic settings	[15, 33, 33–44]	14	2
Gender determination in clinical forensic settings	[15–17, 45]	4	2
Assessment and management of risk and violent reoffending among prisoners	[46]	1	2
Bruise dating in clinical forensic settings	[47]	1	2

## Discussion

This review aimed at identifying the AI models used by forensic pathologists or physicians in their daily practices thanks to a systematic search of the AI applications intended for medicolegal practice and described in the literature. This search resulted in the identification of 378 articles from reference databases and the inclusion of 35 studies published between 1999 and 2022. For each study, the level of integration or maturity of each AI application was assessed in order to map the current medicolegal practices involving AI. The information extracted from the included reports showed that AI is developing in thanatology and clinical forensic medicine (see Table 5). In thanatology, AI models were designed for postmortem identification, the determination of the causes of death, and the estimation of the postmortem interval. In clinical forensic medicine, AI was used to estimate the age of living individuals, the risk of violent reoffending among prisoners and bruises dating. In [15, 16], and [17], an AI model was developed both for age estimation and gender determination. However, the final field of application of the AI models was not clear, that is to say that the expertise field in which the model is expected to be used was ambiguous. For instance, in [36] and [17], the AI model may be used for postmortem identification or age estimation in forensic clinical settings. Therefore, in this review, it was assumed that, when the final field application was not clear, if the model may be applied to several expertise fields, those fields were considered as application fields of the model.

It is worth mentioning that the included articles did not explicitly report any AI application that is currently used by forensic pathologists or physicians in daily practice to date. Therefore, the AI applications appeared to be still in research and development stages. Since the application of AI in forensic medicine is subject to a recent renewal of interest in forensic medicine, as suggested by the publication date of the articles, it may be too soon to observe AI applications in medicolegal routine. This result may also be due to a low model performance or common AI-based issues.

Model performance is summarized for each AI application in Table 4. Currently, there is no well-defined threshold above which model performance is considered high enough to use the model in production. Moreover, this threshold should differ depending on the AI application. However, a model that performs worse than non-AI methods described in the literature or gold standards may be considered as a low-performance model. The comparison of model performance with the non-AI methods by expertise field is given in Table 6. No numerical comparison of performance with gold standard methods was made in 27 reports, a similar or lower performance is found for 3 reports, and models outperform non-AI methods in 5 reports. The performance of the models and their comparison to non-AI methods is quantified in Table 7. Articles that did not provide a quantified comparison between the performance of the AI model and the performance of non-AI methods often compare the performance with previous studies in which other AI models were developed. In order to assess the relevance of a model to apply in medicolegal routine, a quantified comparison of model performance between the AI and gold standard method should be provided. Ideally, the performance metrics should be compared from the same dataset to avoid epistemic variations.

**Table 6:** Comparison of model performance with non-AI methods described in the literature for each included report

Expertise field	No relevant numerical comparison of performance	Similar or lower performance	Better performance
Postmortem identification	[13–15]	[18, 19]	[16, 17]
Postmortem interval estimation	[20–22]	-	-
Determination of the causes of death	[23–31]	-	-
Age estimation in clinical forensic settings	[15, 32, 33, 37, 37–41, 43, 44]	[42]	[34, 35, 46]
Gender determination in clinical forensic settings	[15, 45]	-	[16, 17]
Assessment and management of risk and violent reoffending among prisoners	-	-	[46]
Bruise dating in clinical forensic settings	[47]	-	-

**Table 7:** Comparison of model performance between non-AI methods and AI models. *AUC*, area under the curve; *MAE*, mean absolute error

Reference	Performance of the non-AI method	Performance of the AI model
Constantinou et al. 2015 [46]	AUC score range from 0.665 to 0.717	AUC score is 0.78
Stern et al. 2016 [34]	Error between 0.65 and 0.72 years	Best MAE is 0.36 ± 0.3 years
Spampinato et al. 2017 [35]	Error is 30% higher than the AI model	MAE is 0.79 years
Milosevic et al. 2019 [16]	Accuracy ranges from 0.71 to 0.95	Accuracy is 0.9687 ± 0.0096
Turan et al. 2019 [17]	Accuracy ranges from 0.807 to 0.901	Accuracy is 0.95
Peleg et al. 2020 [18]	Accuracy is 0.845 for Australian and 0.865 for African American	Accuracy is 0.863 for European American, 0.82 for Israeli, and 0.816 for African American population
Peña-Solorzano et al. 2020 [19]	Error in the range of 1 to 11 mm	MAE is 2 mm
Vila-Blanco et al. 2020 [42]	Best median and mean error are − 0.02 ± 0.71 and − 0.04 years respectively. Best MAE is 0.488 years	Median and mean error are − 0.01 ± 0.8 and − 0.04 years respectively. MAE is 0.72 years

However, model performance should not be interpreted as is, since models may show good performance for a given dataset but may be biased towards the validation set. Thus, the model performance must be assessed with a test set in order to prevent biases [48]. A test set was used in approximately 58% of articles. However, no test set was used in 14 articles [13, 14, 17, 20, 24, 25, 32, 33, 35, 36, 38–41]. Therefore, model performance in those articles should be interpreted with caution.

Moreover, despite a good performance on the test set, a model may not be able to generalize to new data. In this case, the model performance may be overestimated due to model overfitting [49]. This issue, common when developing machine learning models, was explicitly handled in 11 reports by techniques based on model architecture [34, 41] and parameters [38, 40, 44, 45], input data [41], and validation steps [20, 43, 46, 47]. In [34] and [41], the model architecture was modified to reduce overfitting by dropout regularization, that is to say removing nodes in a model by a given probability in order to simplify it. Moreover, the authors in [41] added batch normalization layers in the model architecture. This technique is known to reduce the generalization error of the model [50]. AI models were also developed by transfer learning, that is to say the use of a pre-trained model which is then adapted for a specific task, such as age estimation [38, 44] or gender determination [45] of living individuals. In [40], the model parameters were frozen in part of the models along the training phase to prevent overfitting. This parameter fixation may only concern weights of batch normalization layers [31]. In [41], the authors also used data augmentation, that is to say an artificial increase of training data by using transformations, such as image rotations and translations for instance. Indeed, increasing the number of training data helps reducing the problem of overfitting in computer vision tasks [51]. To monitor the effect of overfitting, the performance of the model for the validation set was computed at given steps [20], all along [43] or at the end of the training phase [43, 47]. In [43], the authors did not compute the model performance only once as in [47] but 10 times by using tenfold cross-validation. This technique involves splitting a dataset into a training set and a validation set 10 times with different instances in the validation set for each fold. This gives rise to 10 datasets of training and validation sets. Then, each dataset is used to train a model on the training set and assess the performance on the validation set independently from the other datasets. Thus, cross-validation enables to monitor the effect of overfitting by comparing the performance of the training set and the validation set. Moreover, this technique enables to calculate a mean and a standard deviation of performance for the 10 validation sets, which leads to better assessments of model performance than the use of a unique validation set. To sum up, several techniques may be used to reduce overfitting when developing a model. However, the number of articles that explicitly defined how overfitting was handled is clearly insufficient. This leads to wonder whether the models are able or not to generalize to new data in articles that did not handle overfitting. Therefore, model overfitting must be studied before any use of AI model in medicolegal routine.

The datasets used to develop a model should also be taken into account when assessing the performance of a model, since that model is trained for input data with specific characteristics. In this review, all the studies clearly defined input characteristics or eligibility criteria, except for [23] and [28] which did not show any restriction on the study population in terms of excluded cases (see Online Resource 2). Thus, the data used to develop models may not be representative enough for a given case. For instance, the authors in [19] took photographs of volunteer’s bruises made by projectiles fired from paintball guns in order to date the resulted injuries. All the volunteers were between 22 and 68 years old. The final model showed good performance metrics (> 96% for precision, sensitivity, and specificity). However, only one volunteer was above 40 years old and all the injuries were located on an arm, a leg, the back, the chest, or a buttock. For those reasons, despite the good model performance, the model may not be able to date bruises from the head, which is a target of injuries in cases of domestic violence for instance [52], or for people aged above 40 years old since it was not well trained on those characteristics. Therefore, the restriction of input data characteristics may prevent the models to be used daily by forensic pathologists or physicians due to non-representative datasets used to develop the models reported in this review. Moreover, in classification models, output data may be imbalanced, that is to say that data categories are over-represented compared to others, which often leads to a good model performance for those over-represented categories at the cost of a low performance on the other categories. From the included studies, data output appeared imbalanced towards age [16, 34, 35, 37, 40, 42–45] and feature classification [19]. Data imbalance may lead to model biases towards the most represented classes [53]. Therefore, model performance assessed from high data imbalance should be carefully interpreted for use in production.

It is worth mentioning that neural networks, a type of AI model that currently requires a high volume of data compared to other types of AI models [54], were developed in 26 reports [14–17, 19, 20, 23–25, 27–31, 34–36, 38–45, 47]. All the dataset size appeared highly variable in the studies, as shown in Online Resource 2. The number of cases used to develop models ranged from 10 [33] to 5756 cases [21]. Nevertheless, the maximal number of cases identified from reports may be higher. Indeed, the authors in [39] used more than 12,000 images to develop their models. However, the authors did not detail the presence of identical sources, that is to say if images come from a same individual. Thus, it was not possible to assess the true number of cases used to acquire those images. Therefore, even though a high number of instances were used to develop models, the number of cases considered should be taken into account since a low number of cases may reflect a lack of data representativeness.

In this review, limitations may be identified at first glance. First, only 3 bibliographic databases were explored (PubMed, Scopus, and ScienceDirect) to select articles of interest. One may wonder if the content which may be retrieved from those databases may not be representative of the current knowledge. However, main if not all forensic journals are indexed in these databases. Second, AI applications are rapidly emerging in forensic medicine. Therefore, this field should be regularly monitored to report the state-of-the-art about the usages of AI by forensic medical pathologists or physicians. Third, the final user of AI models was not always obvious and that user may be a specialist such as an anthropologist for models designed from bone-related data analysis or a psychologist for behavior-based algorithm. Moreover, the daily tasks of a forensic pathologist or physician may differ from one country to another, thus making it difficult to determine the final user of a model.

However, this review enables to maintain a good overview of the use of AI applications in forensic medicine through time. First, it reports a state of the art of AI applications used by forensic pathologists and forensic physicians. Second, this review also reports the levels of integration of each AI models included, which enables to follow the evolution of AI applications from the concept to their use in medicolegal practice. Therefore, this review may later report a history of AI applications developed for forensic medicine purposes. Finally, the search equations (see Table 1) enable to easily extract the articles of interest and update the review regularly in order to report the future usages of AI in forensic medicine.

To sum up, the analysis grid given in Table 2 and derived from the TRIPOD checklist enabled us to analyze several aspects of predictive models development in the articles, such as the input data characteristics or the model performance. All the features described in the TRIPOD checklist enable to provide transparency regarding the model development process. Globally, all the articles described TRIPOD features but only 5 articles seemed to follow the TRIPOD guidelines completely without any lack of critical information. However, 30 articles lacked at least one feature, critical or not, from the TRIPOD guidelines such as a clearly defined distribution of datasets or the number of participants in a study, which prevent any complete assessment of model applicability. Therefore, if a model development process does not provide enough information or does not report explicitly or correctly any critical criterion given in Table 2, the resulting model could not be directly transposed to a medicolegal routine use.

It is worth mentioning that the model performances described in the articles are highly heterogeneous with a majority of articles highlighting good model performance, which suggests that models may perform well in daily practice. However, when diving deeper into the model development process, one may notice that the models may not be applicable to medicolegal practice due to several factors, such as real cases meeting one or several exclusion criteria of the sample or population of study for instance. Moreover, the apparent lack of data to develop or validate AI models in the corpus of articles is hurdle to the application of models in daily practice, since such lack would not provide sufficient confidence or reliability to use those models. Furthermore, it may be difficult to understand how advanced AI models, such as dense neural networks, make decisions or predictions, so that they may be perceived as black box models. The use of such models in routine may thus be unwanted by forensic pathologists or forensic physicians due to a lack of model explainability or understanding. For all those reasons, model performance is clearly not sufficient to assess model applicability. Besides, the raw performance of algorithms, even evaluated only in laboratory conditions and not yet confronted with the reality of daily practice, must always be looked at from several aspects. First, performance must be evaluated according to several complementary criteria or metrics and never by just one. There is no single criterion to account for the performance of an algorithm. Then, these performances must be confronted with the existing one: does the algorithm do better than what we are currently doing? Finally, this “better,” when it exists, must be studied according to several components. The first is similar to what is called “clinical significance” in the case of classic trials in medicine, for example, for the evaluation of a drug or an intervention. The “statistical significance” weighs nothing against the need for a really and sufficiently increased utility to justify a change of tool or practice. Indeed, if a new algorithm displays a performance of 82% compared to a well-proven, reliable, and installed practice with a performance of 81%, switching from one to the other is not obvious and is not necessarily justified. Other important aspects must be taken into account, such as the modifications either necessary (e.g., new equipment, software change, new data collection, data regulation) or induced by the adoption of this algorithm in current practice.

To this criterion of clinical significance must be added the ethical nature of the use of algorithms. On the one hand, we must keep in mind that these algorithms are developed from data, and that their quality cannot exceed that the quality of the input data. Worse, the use of a biased algorithm tends to reproduce and then reinforce these biases. Biases of gender, age, ethnic origin, and socio-economic level already present in the majority of classic clinical and epidemiological studies are now incorporated into the algorithms. On the other hand, the fact of using an algorithm does not exonerate from keeping in mind, depending on the field of application, what a rate of false positives or false negatives represents the following: as efficient as it is, do we want to ethically take a greater or lesser risk of falsely concluding that an isolated minor is older than 18, or that a third party is involved in a criminal act?

The impact of the adoption of AI by forensic pathologists does not stop at their personal practice of medicine. Indeed, like the use of new techniques such as DNA or neuroimaging in criminology, the introduction of these new more or less autonomous, more or less normative, and biased decision-making tools is and will be examined by the other stakeholders, starting with lawyers and magistrates. The full adoption and acceptability of AI in forensic medicine are therefore also conditional on acceptance by these stakeholders.

Finally, we must not neglect the very practical side of the introduction of AI into our daily practice. In order to be able to use algorithms in the most fluid, secure and reliable way, it is necessary that they can be integrated into a work environment that allows it. In concrete terms, this means, for example, that there is already a suitable information system and quality data collection compatible with the use of AI, as well as practitioners trained in this entire necessary data chain. However, we are generally not very far from it. A general convergence of tools and practice is therefore necessary. More broadly, it seems important to us that scientific societies, national and international, take up this subject of data and AI, and be able to formulate recommendations and guidelines to good practice concerning their use.

## Conclusion

In forensic medicine, the AI applications meant to be used by forensic pathologists or physicians in daily practice are mainly intended to thanatology and clinical forensic medicine purposes. The main expertise fields in which AI applications are developed are postmortem identification, the determination of the causes of the death, the estimation of the postmortem interval, and the estimation of the age of living individuals. However, according to the literature, no AI application seems to be daily used by forensic medical doctors since the AI models remain in research and development stages. This may be explained by low or overestimated model performances, a lack of representative datasets, or the introduction of biases into AI models. Moreover, the implementation of AI in medicolegal practice does not only concern forensic pathologists or physicians but also magistrates and barristers since medicolegal expertise is intended for justice institutions. Therefore, AI should be appropriated by forensic pathologists and physicians as well as legal professionals to be integrated in forensic medicine practices.

### Supplementary Information

Below is the link to the electronic supplementary material.

Supplementary file1 (PDF 335 KB)

Supplementary file2 (PDF 248 KB)
