# Supplementary material for: Artificial intelligence in the practice of forensic medicine: a scoping review
Source: Int J Legal Med. 2023 Dec 13;138(3):1023–37. doi: 10.1007/s00414-023-03140-9 (PMC11003914; doi:10.1007/s00414-023-03140-9)
Supplement: Supplementary file 1 — Supplementary file1 (PDF 335 KB) [file 414_2023_3140_MOESM1_ESM.pdf]

Online Resource 1

Article title: Artificial intelligence in the practice of forensic medicine: a scoping review

Journal name: International Journal of Legal Medicine

Author names: Laurent Tournois, Victor Trousset, Didier Hatsch, Tania Delabarde, Bertrand Ludes, Thomas Lefèvre

Corresponding author: Laurent Tournois
Université de Paris Cité, CNRS UMR 8045, F-75006 Paris, France
BioSilicium, Riom, France.
Mail: laurent.tournois@biosilicium.fr

Full results of sources of evidence. AI: artificial intelligence, ANN: artificial neural network, AUC; area under the curve, BA: biological age, CA: chronological age, CNN: convolutional neural network, CV: cross validation, FN: false negative, FNR: false negative rate, FP: false positive, FPR: false positive rate, HP: hyperparameter, IoU: intersection over union, KCRD: Kütahya Child Radiology Dataset, kNN: k-nearest neighbors, LR: likelihood ratio, MAE: mean absolute error, MLP: multilayer perceptron, MRI: magnetic resonance imaging, OPG: orthopantomogram, PMCT: postmortem computed tomography, PMI: postmortem interval, RBFN: radial basis function network, RMSE: root mean square error, SEE: standard error estimate, TN: true negative, TNR, true negative rate, TP: true positive, TPR: true positive rate, t-SNE: t-distributed stochastic neighbor embedding.

Reference	Publication type	Publication reliability	Outcome	Data sources	Population / sample study	Input data	Model development	Performance metrics	Model evaluation	Real application	aTRL
Karasik et al., 1999 [12]	Original article	Peer-reviewed article published in 1999	Chronological age (in years)	Real data, living human subjects	Population from Chuvasha (Russia, 293 males and 254 females) and Turkmenia (257 males and 386 females). Subjects' age ranging from 17 to 86 years old. Exclusion criteria: bone disease, steroid medicine use, post-traumatic,	Only a training set (no validation or test set). Features: osseographic score, osteoarthritis (OA) score and osteoporosis (OP) score. No OA score for 7 Russian subjects (instances removed depending on the AI model)	Multilinear regression. Equations are given.	R^2 and standard error estimate (SEE)	SEE ranges between 4.9 to 6.25 years for the best models, R^2 between ground truth and estimated values range is 0.818 to 0.901	No use reported in real case	2

					rheumatoid, psoriatic osteoarthrisis or contractures due to tenosynovitis of the palmar flexors						
Karasik et al., 2000 [13]	Original article	Peer-reviewed article published in 2000	Chronological age (in years)	Real data, living human subjects	5 756 living individuals (2683 males and 3073 females) belonging to 9 ethnic groups (mostly rural autochthonous populations) Subjects' age ranging from 17 to 96 years old. Exclusion criteria: bone disease, steroid medicine use, post-traumatic, rheumatoid, psoriatic osteoarthrisis or contractures of the palmar flexors	Only a training set (no validation or test set). Feature: osseographic score. Missing 6 females, no information about how missing data is handled. Possible sampling bias due to population characteristics (declared by authors)	Logistic regression. Equations are given.	R^2 and standard error of estimate (SEE) for each ethnic group and each sex group	SEE ranges between 4.22 to 6.64 years for the best models, R^2 between ground truth and estimated values range is 0.671 to 0.901. Weak extrapolation ability of logistic formula from one ethnic group to another one.	No use reported in real case	2
Bocaz-Beneventi et al., 2002 [14]	Original article	Peer-reviewed article published in 2002	PMI in hours	Real data, deceased human subjects	61 cases. PMI between 7 and 144 hours	Train set (51 cases) and validation set (10 cases). No external validation. 8 features corresponding to NH_4^+, K^+, Na^+ and Ba^{2+} peak area and heights.	ANN and linear least squares regression. ANN Architecture and regression equation are given. Overfitting reduced by assessing the model performance with the validation set every 100 or 1000 epochs.	HP tuning assessed with RMSE. Performance on the validation set assessed by the correlation coefficient between predicted and ground truth PMI values.	Correlation coefficient between predicted and real values is 0.9810	No use reported in real case	2

Constantinou et al., 2015 [15]	Original article	Peer-reviewed article published in 2015	Risk of violence (yes, no) revised from 5 variables (acquisitive crime convictions, age, gender, violent convictions and PCLR score)	Real data, living human subjects	953 cases (778 males and 175 females). Prisoners serving sentences (for sexual or violent offences) for at least 2 years. Missing data is inferred from evidence provided from other factors within the model and linked to the missing data.	Only 1 dataset used for 10-fold CV. No external validation. All features are explicit (n = 89)	Bayesian network with binary predictions. Architecture of network is given. Overfitting is estimated by comparing AUC with and without 10-fold CV.	AUC	Best AUC is 0.78 (10-fold CV). Performance too low for a use in medicolegal practice	No use reported in real case	2
Simmons et al., 2016 [16]	Original article	Peer-reviewed article published in 2016	Human or non-human bone	Real data, deceased human and animal subjects	6 bones from humans, 7 bones from animals	Only a training set (no validation or test set). Features: tissue classification system used by Cuijpers [55].	Decision tree, architecture is described.	Accuracy	Accuracy is 1	No use reported in real case	2
Stern et al., 2016 [17]	Conference paper	Not peer-reviewed article and published in 2016	BA in years	Real data, living human subjects	Male Caucasian aged between 13 and 25 years old.	240 hand MRI images splited into 8-fold CV with 30 subjects for model validation and the remaining 210 subjects (data augmentation up to 1050 images) for model training. Images are directly used as inputs.	Deep CNN. Architecture is described. Loss function takes into account BA or CA. Overfitting is handled by dropout regularization (dropout ratio is 0.5)	MAE for BA estimation and TPR, FPR, TNR, FNR for majority classification (above 18 years old)	Best MAE is 0.36 ± 0.3 years with training with BA and 0.56 ± 0.44 years compared with training with CA. Best TPR, FPR, TNR and FNR obtained with training with CA for CA > 18 years old are respectively 98.7, 3.6, 96.4, 1.3. For classification with BA > 18 and training with BA, those values are respectively 100, 0, 100, 0	No use reported in real case	2
Yilmaz et al., 2017 [18]	Technical note	Peer-reviewed article published in 2016	Live or stillbirth	Real data, deceased human subjects	44 cases (24 live and 20 stillbirth).	Training set (77% of data) and test set (23% of data). Features are explicit (n = 10). Missing data is	MLP, logistic regression and RBFN (Architectures are given).	Specificity, sensitivity, F-score, accuracy. MAE and RMSE are used to assess	ANN and RBFN are the best models. Best specificity is 0.833, sensitivity is 1, F-score is 0.9091, accuracy is 0.9, MAE	No use in real case is reported	2

						removed		the model performance on the test set but the computation of the type of error is not clear.	is 0.1149 and RMSE is 0.3060.		
Ebert et al., 2017 [19]	Original article	Peer-reviewed article published in 2017	Presence of hemopericardium and localization of blood within the pericardium	Real data, deceased human subjects	52 subjects (34 males and 18 females).<br>Exclusion criteria: advanced decomposition, thoracic trauma, evidence of blunt or sharp force injury, presence of cardiac medical devices, hemothorax	Train set (50 % of data) and test set (50% of data) generated 20 times using a different randomization approach.<br>No external validation.<br>Data unbalanced in gender.<br>Feature: PMCT images	ANN (architecture is not given)	Precision, recall and F-score.<br>The meaning of those metrics for the localization task is not clear.	Presence of hemopericardium: average precision, recall and F-score are respectively 0.85 ± 0.11, 0.77 ± 0.26, 0.77 ± 0.16.<br>For the localization task, those values are respectively 0.79 ± 0.05, 0.78 ± 0.05, 0.78 ± 0.0003	No use in real case is reported.<br>AI models must be further validated (the article only deals with a feasibility study).<br>Full integration of the AI models in the picture archiving and communication system is required for a use in daily routine. No challenge for the forensic pathologist to detect hemopericardium in PMCT images.	2
Spampinato et al., 2017 [20]	Original article	Peer-reviewed article published in 2017	Bone age in years	Real data, living human subjects	1391 cases from the Digital Hand Atlas Database System [56].<br>Subjects' age is lower than or equal to 18 years old.	Train and validation sets created by 5-fold CV.<br>No external validation.<br>Data is balanced in gender and race (Asian, black, Caucasian and Hispanic) but unbalanced in age.<br>Feature: left hand X-ray image	CNN with regression network.<br>A bias towards age is highlighted.	MAE	Best MAE is 0.79 years (mean of 2 readings as ground truth). MAE varies between 0.35 years (for Asians males between 0 and 9 years old) and 1.16 years (for Caucasian males between 0 and 9 years old)	No use in real case is reported.	2
Stern et al., 2017 [21]	Conference paper	Peer-reviewed	CA in years	Real data,	103 Caucasian male volunteers	Train and validation sets (4-	Random forest and CNN	Matrix confusion (TP,	Best accuracy is 91.3%, sensibility is	No use in real case is reported	2

		article published in 2017		living human subjects	aged between 13 and 25 years old. 309 3D MRI images	fold CV). No external validation. Uniform distribution in age.	(architectures are given but not detailed)	TN, FP, FN), Accuracy, Sensibility, Specificity (for the classification in terms of majority age), MAE for CA estimation)	0.886, specificity is 1, MAE is 1.14 years. Best performance with CNN but authors declared to be careful with distinction between majority and minority age		
Zhang et al., 2018 [22]	Original article	Peer-reviewed article published in 2017	Skeletal age in years	Real data, living human subjects	562 Chinese subjects without history of chronic illness, trauma, physical deformity, surgical procedure that might affect stature or sternum dimensions. Subjects' age ranging from 20 to 90 years old.	512 cases in the training set and 50 in the test set. Data is balanced in gender, unbalanced in age. Features: average radiation density of upper, medial, lower of the first costal cartilage and stages of ossification of costal cartilage from left and right sides.	Linear regression (simple and multiple), SVM, decision tree, gradient boosting Architecture and equations are given.	MAE, error range, least absolute error, proportion of correct predictions within 5 years and within 10 years	MAE is 5.31 years for males and 6.72 years for females.	No use in real case is reported	2
Canturk et. al., 2018 [23]	Original article	Peer-reviewed article published in 2018	PMI interval (20, 40, 60, 80 and 100 min intervals)	Real data, deceased human subjects	10 subjects from Istanbul (1 female, 9 males) aged between 30 and 70 years old without corneal opacity affected by cause of death or prone position, no head or cervical trauma or edema. 450 images of the eye (45 images per subject)	Train and validation sets (10-fold CV) or 9 subjects in the train set and 1 in the test set. No external validation. Balanced data. Features are explicit (n = 61)	Linear and radial basis function (SVM) and kNN	Accuracy	Best performance for SVM. Accuracy ranges from 0.715 to 0.89 (depending on the time interval)	No use in real case is reported	2
Heimer et al., 2018 [24]	Original article	Peer-reviewed	Presence of fracture or intact	Real data,	75 cases with skull fractures	Train and validation sets (2-	Deep neural network (the	AUC, sensitivity,	Best AUC 0.965, sensitivity 0.914,	No use in real case is reported	2

		article published in 2018	skull	deceased human subjects	and 75 cases without fracture. Subjects' age ranging from 18.96 years old to 95.6 years old. Fractures come from accidents, suicides, unknown manner of death, one criminal offense. Controls come from natural death followed or not by accidents and unknown. Exclusion criteria: age < 18 years old, shattered skulls lacking resemblance to physiological anatomy, visible residues from surgical intervention	fold CV) generated 100 times with random sampling. No external validation. Data imbalance in gender (male-to-female ratio is 70/30) and manner of death (number of instances varies between 1 and 55 depending on the manner of death). Feature: Head PMCT image.	architectures are not described).	specificity	specificity 0.875 (classification threshold is 0.79)		
Koterova et al., 2018 [25]	Original article	Peer-reviewed article published in 2018	Age at death in years (no detail about BA or CA)	Real data, deceased human subjects	941 samples. Subjects' age ranging from 19 to 100 years old.	Train and validation sets (5-fold CV). Data unbalanced in race (Caucasian, Afroamerican, African and Asian) and balanced in gender. Features: measurement from the pubic symphysis and the sacro-pelvic surface	ANN, decision tree, M5 tree (decision tree with linear regression function at the leaves), kNN, multilinear regression, collapsed regression model. Architectures are not always given.	MAE, RMSE	Best performance for M5 tree and multilinear regression (MAE is 9.7 years and RMSE 13.3 years). The performance is similar between males and females.	No use in real case is reported	2
Matoba et al., 2018 [26]	Original article	Peer-reviewed article	Lung weight in grams	Real data, deceased	111 deceased subjects (222 samples, 2	No information about the datasets used.	Multivariate linear regression (the	R^2 between lung weight measured	R^2 is 0.89. The model is not applicable if lung	No use in real case is reported	2

		published in 2018		human subjects	samples per subject). Exclusion criteria: severely corrupted, unconfirmed lung weight, more than 6h between PMCT and autopsy, severe putrefaction. Subjects' age ranging from 18 to 95 years old, PMI ranging from 0.3 to 60 days.	Unbalanced data in cause of death. Features: 6 variables corresponding to interval of HU volume in mL	equation is given).	during autopsy and predicted lung weight.	weight < 300 g		
Stern et al., 2019 [27]	Original article	Peer-reviewed article published in 2019	Biological age (BA) or chronological age (CA)	Real data, living human subjects	328 3D hand MRI or 835 2D x-ray images. 3D MRI dataset: Caucasian male volunteers (aged between 18 and 25 years old), 141 males are under 18 years old, no history of endocrinal, metabolic, genetic or developmental disease 2D X-ray dataset: subjects from the Digital Hand Atlas Database [57] aged between 10 and 19 years old	Train and validation sets (4-fold CV). Uniform distribution in age for 2D dataset. No external validation. Features: 13 cropped bone images.	CNN. The architecture is described. Overfitting is reduced by transfer learning.	MAE and AUC	3D dataset: MAE is 0.2 ± 0.42 years (for BA estimation), 0.82 ± 0.65 years (for CA estimation), AUC 0.9567 (for the distinction between the majority and minority age). 2D dataset: MAE 0.58 ± 0.61 years (for BA estimation), 0.83 ± 0.66 years (for CA estimation)	No use in real case is reported	2
Andersson et al., 2019 [28]	Original article	Peer-reviewed article published in 2019	LR between PMI intervals.	Real data, deceased human subjects	101 cases. Swedish indoor settings. Subjects without presence of insect activity, no major traumatic	Train set (93 cases) and test set (8 cases). Features: partial body scores for head, trunk and limbs.	Bayesian network (architecture is given).	LR	LR < 1	No use in real case is reported	2

					damage, no submersion, no burn.	Missing values or values deemed potentially biases are assigned a null value.				
Avuclu et al., 2019 [29]	Original article	Peer-reviewed article published in 2019	Tooth age in year intervals (4-9, 10-14, 15-22, 23-63 years old) and gender (male, female)	Real data, human subjects	162 different age groups from 4 to 63 years old.	1 315 teeth images. Train set (size is not given) and test set (size < 12 images). Features: mean of pixels values (pixel value - mean of image pixel value) for each sub-segment of an image	MLP. The architecture is given.	Difference between predicted and true age for age estimation and classification success for gender determination	Age estimation: difference between 0 and 6 years. Gender determination: success rate between 2.5% and 100%	No use in real case is reported	2
De Back et .al, 2019 [30]	Conference paper	Peer-reviewed article published in 2019	CA in months	Real data, living human subjects	Subjects' age ranging from 5 to 25 years old.	More than 12 000 OPG. Train set (75% of data) and validation set (25% of data). Small dataset sizes. No external validation and no mention of HP tuning. Feature: OPG (image).	Bayesian CNN (architecture is given)	MAE and concordance correlation coefficient between true and predicted CA	Overall MAE is 21 months, concordance correlation coefficient between true and predicted CA is 0.910 on 2400 images (validation set). Lowest MAE is 12.8 months for 50 to 75 months old and highest MAE is 28.6 months for 275 to 300 months old.	No use in real case is reported	2
Li et al., 2019 [31]	Original article	Peer-reviewed article published in 2019	CA	Real data, living human subjects	1 875 participants from the West China Han group and aged between 10 and 25 years old. Exclusion criteria: evident deformities or disease in the pelvic region	1498 images for training and 377 for test. Balanced data in gender. Age groups of 1-year intervals. Slight imbalance in age (more than twice as much as individuals in certain age groups than others for train and test datasets except test	CNN (architecture is described). Overfitting is handled by freezing the convolutional layers of the model.	MAE and RMSE.	Mean MAE is 0.94 years, mean RMSE is 1.30 years. No statistical difference between males and females. Highest MAE is obtained for 24-25 years old subjects and best MAE is obtained for 10-11 years old subjects. MAE ranges between 0.11 and 2.71 years. RMSE ranges	No use in real case is reported	2

						females). Images with superposition abdominal organs over the iliac crest removed from train set but kept in test set. No external validation. Feature: pelvis X-ray radiograph			between 0.15 and 2.57 years. Those range limits are obtained for females (males are in between).		
Milosevic et al., 2019 [32]	Conference paper	Peer-reviewed article published in 2019	Male or female	Real data, living human subjects	European (Caucasian) subjects aged between 19 and 85 years old (female-to-male ratio: 58.8/41.2)	4 000 OPGs (number of subjects not given), images may come from the same subject. Train and validation sets (77% of data) and test set (23% of data). 10-fold CV for performance evaluation on all the images (train, validation and test datasets gathered). Data unbalanced in age. Feature : OPG (image)	CNN (architecture is given)	Accuracy	Accuracy is 0.9687 ± 0.0096	No use in real case is reported	2
Turan et al., 2019 [33]	Original article	Peer-reviewed article published in 2019	Male or female	Real data, living human subjects	284 subjects aged between 24 and 60 years old, without operation, subluxation, bone fracture or deformities.	Train set (80% of data) and validation set (20% of data) shuffled at each iteration. No external validation. Features: 8 anthropometric measurements of bone length of the first and fifth phalanges and	MLP (architecture is given)	Sensibility, specificity, accuracy, Matthews Correlation Coefficient (MCC)	Best accuracy is 0.965, specificity is 0.973, sensitivity is 0.956 and MCC is 0.929. Variance is high suggesting a probable overfitting	No use in real case is reported	2

						metarsals measured times and averaged per subject (mean and standard deviation of each feature is provided). Data is balanced in gender.					
Abderrahmane et al., 2020 [34]	Conference paper	Peer-reviewed article published in 2020	CA in years	Real data, living human subjects	190 subjects aged between 18 and 75 years old.	11 076 hand photographs. Train set (70% of data) and validation set (30% of data). Data is balanced towards age after balancing (ages are 18 to 26, 28 to 30, 36, 43, 54, 70, 75 years old). About 710 images per age subset. No external validation. Data imbalance in age, gender, skin triplet. Feature: hand photograph.	CNN combined with gated recurrent units (architecture is given). Overfitting is handled by balancing data with data augmentation and by using batch normalization and dropout layers.	MAE	Learning curve highlights underfitting (validation loss is under lower than the training loss). MAE is 2.373 years. With skin color and gender adjustment, MAE is 1.9266 years.	No use in real case is reported	2
Garland et al., 2020 [35]	Original paper	Peer-reviewed article published in 2020	Presence of fatal head injury (yes, no)	Real data, deceased human subjects	50 subjects (25 cases with fatal head injuries, 25 suicide hanging deaths). Transport related and accidental fatal deaths for cases with fatal head injuries. Exclusion criteria: suspicious, homicidal and deaths of children aged less than 10	Train set (40 cases: 20 cases with fatal head injuries and 20 controls) and test set (10 cases: 5 cases with fatal head injuries and 5 controls). Validation on 20% of the training set. Data unbalanced in gender (male-to-female ratio is 19/6 for cases	CNN (architecture is not given)	Accuracy	Accuracy is 0.7	No use in real case is reported	2

					years old due to potential legal issues, signs of decomposition, cases with neurosurgical procedures.	with fatal head injuries, 22/3 for controls). Feature: head PMCT images					
Homma et al., 2020 [36]	Conference paper	Peer-reviewed article published in 2020	Drowning or non-drowning death	Real data, deceased human subjects	280 cases: 140 drowning (3 784 images) and 140 non drowning (3 863 images) cases	Train and validation sets (10-fold CV with balanced data in drowning distinction and same size for each fold). Feature: lung PMCT image.	CNN (architecture is given)	AUC	AUC is 0.879	No use in real case is reported	2
Peleg et al., 2020 [37]	Original article	Peer-reviewed article published in 2019	Male or female	Real data, living human subjects	461 subjects. No subject with more than 2 absent ribs, no measurements from broken or deformed ribs (train dataset)	Train set (413 subjects, European Americans and African American, aged between 20 and 87 years old, unbalanced data in gender), leave-one-out CV set (33 adults aged between 10 and 60 years old, race is not provided, balanced data in gender), 15 adult for validation of virtual measures (race is not given). Features: anthropometric measures of the ribs and the sternum. Instances with missing data are kept if ribs are missing (models are applicable for	Multivariate linear regression (equations are given)	Success rate (not clearly defined)	Success rate ranges from 0.667 to 0.89	No use in real case is reported	2

						individual ribs)				
Pena-Solorzano et al., 2020 [38]	Original article	Peer-reviewed article published in 2020	Localization of the femur and classification of orthopedic implants in the femur	Real data, deceased human subjects	450 subjects aged between 20 and 90 years old. Inclusion criteria: only males, cause of death due to a natural disease or drug overdose (to avoid physical trauma cases but there are some)	Train set (70% of data), validation set (15% of data) and test set (15% of data) with 5-times random subsampling for the localization task and 8 times for the classification task. Imbalance in class sizes for the classification task. Feature: PMCT image (with the subject's age for the classification task)	Residual networks (presence or absence of femur). Hybrid convolutional autoencoder (feature extraction) + kNN for t-SNE classification of absence of implant, nail, hip replacement and knee replacement. Architectures are described.	Localization of the femur (test): MAE, Jaccard similarity coefficient (= IoU), Dice score (= F1 score). Classification task: accuracy, precision, recall, F1 score	Best results for femur localization: MAE between 0 and 13.1 mm, Dice between 1 and 0.93, IoU between 0.91 and 1 depending on the CT plane. Depending on the class to predict for the classification task: accuracy between 0.97 and 1, precision between 0.91 and 0.99, recall between 0.65 and 1, F1 score between 0.76 and 0.98	No use in real case is reported	2
Tirado et al., 2020 [39]	Technical note	Peer-reviewed article published in 2020	Bruise date estimate in time intervals	Real data, living human subjects	11 subjects (4 females and 7 males) age between 22 and 68 years old. Bruises result from paintball impacts.	Train set (1 712 instances), validation set (215 instances) and test (213 instances). 10-fold CV is used on train. Data unbalanced in bruise location and age. Data balanced between validation and test set but unbalanced for each class (including the train dataset) Feature: bruise photograph	CNN (architecture is not given). Early stopping on validation accuracy with patience 3, overfitting is evaluated by the validation precision metric calculated at the end of training (the use of accuracy and precision is not clear)	Sensitivity, specificity, precision, confusion matrix	Best sensitivity and precision are 0.97 and specificity is 0.995.	No use in real case is reported	2
Vila-Blanco et al., 2020 [40]	Original article	Peer-reviewed article published in 2020	CA in days	Real data, living human subjects	2 289 Spanish Caucasian subjects aged between 4.5 and 89.2 years old.	8-fold CV with test as held-out set and train and validation sets as the 7 other sets (train 80%, val	CNN (architecture is described)	Age estimation: R^2 between estimated and ground truth age, median	Best R^2 is 0.9 on the whole dataset and on age < 25 years old, however R^2 is 0.53 for ages between 14 and 21	No use in real case is reported. The AI model performance is similar or lower than the	2

						20%). Unbalanced data in age (963 cases for 10-19 years old and 31 cases for 70-89.5 years old). More females than males (> 20%). Feature: OPG image		error and absolute error. Sex classification: accuracy, sensitivity, specificity and AUC. Performance measured on the test set and on ages < 15, 20, 25, 30, 40 years old.	years old. Best accuracy is 0.854 (whole dataset), sensitivity is 0.878 (whole dataset), specificity is 0.845 (age < 40 years old) but 0.823 for the whole dataset, AUC is 0.926 (age < 40 years old) but 0.925 for the whole dataset. For age < 20 years old: the best R^2 is 0.89, accuracy is 0.8, sensitivity is 0.8, specificity is 0.801 and AUC is 0.888.	performance of non-AI models.	
Mauer et al., 2021 [41]	Original article	Peer-reviewed article published in 2021	CA in years	Real data, living human subjects	Caucasian males with middle to high socio-economic status, raised in Hamburg (Germany) or surroundings, aged between 13 and 21 years old, and no chronic diseases or severe bone Injuries.<br>Coronal dataset: 79 male Caucasian subjects aged between 14.41 and 21.66 years old.<br>Sagittal dataset: 297 male Caucasians subjects aged between 13 and 21.83 years old.	Coronal dataset: 2 220 images splitted into train (66%), validation (18%) and test sets (19%, stratified 5-fold CV).<br>Sagittal dataset: 404 images.<br>Data unbalanced in age (age-stratified data augmentation).<br>Missing data us removed if bone info < 2%.<br>Feature: MRI images (for feature extraction and age estimation) with ossification maturity stages and anthropometric data (when used).	CNN + tree-based machine learning algorithm (architecture is given).<br>Overfitting is assessed by a learning curve (training and validation loss values along epochs)	MAE (age estimation), sensitivity, specificity, accuracy and AUC (distinction of the majority age).	MAE is 0.71 ± 0.55 years for the coronal and 0.81 ± 0.62 years for the sagittal dataset.<br>Best accuracy, sensitivity, specificity and AUC are respectively 0.875, 0.884, 0.886, 0.943 for the sagittal and 0.857, 0.864, 0.846, 0.908 for the coronal dataset.	No use in real case is reported	2
Ozdemir et al.,	Original	Peer-	Bone age in	Real	KCRD dataset:	Datasets split into	CNN	MAE, RMSE, R^2	KCRD Dataset: best	No use in real case	2

2021 [42]	article	reviewed article published in 2021	months or year (not clear)	data, living human subjects	5305 hand-wrist radiographs from hospitals in Kütahya of people between 0 and 18 years old (mean 140.33 months). Exclusion of 0-7 month old subjects RSNA dataset: 12611 radiographs of individuals with mean age 127.32 months	training, validation and test sets with proportion 0.7/0.15/0.15 respectively. Data unbalanced in age. Feature: hand-wrist radiogaphs	(architecture is given). Overfitting is handled by transfer learning		MAE, RMSE and R^2 are 4.3, 5.76 and 0.99 respectively. RSNA dataset: best MAE, RMSE and R^2 are 5.75, 7.42 and 0.96 respectively. The units of are not clear.	is reported	
Oura et al., 2021[43]	Original article	Peer-reviewed article published in 2021	Gunshot distance (categories: control, contact, close range and distant)	Real data, dead piglet subjects	Piglet weights range from 2.05 to 4.76kg, piglets died from natural death and are stored 5 days maximum. No overlap of gunshot wounds. Exclusion criteria: external deformability and abnormal or blotchy skin pigmentation	Dataset is composed of 60 negative controls, 50 contact shots, 49 close-range shots and 45 distant shots images. Dataset split into training, validation and test sets with proportion 0.6/0.2/0.2 respectively. No external validation. Feature: photographs of gunshot wounds	MLP (architecture is given). Overfitting not studied but authors assert that applicability to human is likely to be poor	Accuracy, recall, precision, F1 score, AUC	Testing accuracy and F1 range from 0.94 to 1, recall from 0.89 to 1, precision from 0.92 to 1 and AUC from 0.99 to 1. Averaged test accuracy is 0.98	No use in real case is reported	2
Garland et al., 2021 [44]	Original article	Peer-reviewed article published in 2021	Classification of heart histology slides into normal heart, old myocardial or acute myocardial infarction	Real data, dead human subjects	Number of cases is not provided. Exclusion criteria: autolysis, marked decomposition, postmortem bacterial overgrowth, processing artifacts, fading	50 images of normal heart slides, 50 images of old myocardial infarction slides and 50 images of acute myocardial infarction slides. Train, validation and test sets with	CNN (architecture is given). Overfitting is not explicitly handled.	F1-score by class to predict and accuracy	Accuracy and F1-scores are equal to 1	No use in real case is reported	2

					of stains, early acute myocardial infarction (1 day old), healing myocardial infarction and other causes of myocardial scarring	108, 15 and 30 images respectively. No label imbalance. No external validation.					
Ibanez et al., 2022 [45]	Original article	Peer-reviewed article published in 2022	Presence of fracture or not	Real data, dead human subjects	55 females with median age of 64 years old and 140 males with median age of 54 years old. Exclusion criteria: signs of advanced decomposition, organ explantation, severe trauma with extensive damage to the corpse such as amputation or exenteration, deviating scanning protocol, no PMCT data, rib fracture present in the volumetric CT data and the autopsy but not visible in the rib unfolding tool or in which the rib defect was in the cartilaginous part of the rib, still under investigation	585 images (255 with rib fractures, 252 without fracture, 78 with old fractures). 5-times split into 2 sets (train/validation and test sets) then 5-fold stratified CV on the train/validation set (85% of all data) split into 344/86 images then test on 77 images (15% of all data). No external validation. Feature: PMCT images	CNN (architecture is given). Overfitting handled by adjusting the weights only on the batch normalization layers	Mean Recall, mean precision, mean F1 score	Recall, precision and F1 (means) are respectively 0.93±0.05, 0.89±0.03, 0.91±0.04	No use in real case is reported	2
Li et al., 2022 [46]	Original article	Peer-reviewed article published	Gender (male or female)	Real data, living human	1226 males and 896 females Chinese Han individuals from	2326 pelvic anteroposterior radiographs Train/validation	CNN (architecture is given). Overfitting	Average Accuracy	Average Accuracy is 0.946 in Chinese Han population and 0.829 in the	No use in real case is reported	2

		in 2022		subjects	the West China Han group without showing any deformity or diseases in the femur region, and aged between 18 and 26 years old.<br>Caucasian population.	(1915 images) and test (207 images) sets.<br>2 test datasets: 361 pelvic radiographs from Han pop (207 from 18 to 26 years old individuals and 154 from 27 to 80 years old individuals) and 50 pelvic radiographs from Caucasian pop (23 from 18 to 26 years old individuals and 27 from 27 to 80 years old individuals).<br>Data unbalanced in age.<br>Feature: pelvic anteroposterior radiographs	handled by transfer learning.		Caucasian population		
