# Supplementary material for: Artificial intelligence in the practice of forensic medicine: a scoping review
Source: Int J Legal Med. 2023 Dec 13;138(3):1023–37. doi: 10.1007/s00414-023-03140-9 (PMC11003914; doi:10.1007/s00414-023-03140-9)
Supplement: Supplementary file 2 — Supplementary file2 (PDF 248 KB) [file 414_2023_3140_MOESM2_ESM.pdf]

Online Resource 2

Article title: Artificial intelligence in the practice of forensic medicine: a scoping review

Journal name: International Journal of Legal Medicine

Author names: Laurent Tournois, Victor Trousset, Didier Hatsch, Tania Delabarde, Bertrand Ludes, Thomas Lefèvre

Corresponding author: Laurent Tournois
Université de Paris Cité, CNRS UMR 8045, F-75006 Paris, France
BioSilicium, Riom, France.
Mail: laurent.tournois@biosilicium.fr

Characteristics of AI input data for the 35 included studies. AI: artificial intelligence, CT: computed tomography, MRI: magnetic resonance imaging, PMCT: postmortem computed tomography

Reference	Number of cases	Population characteristics	Datasets	Missing data
Karasik et al., 1999 [12]	796 living human individuals	Population from Chuvasha (Russia, 293 males and 254 females) and Turkmenia (257 males and 386 females). Subjects' age ranging from 17 to 86 years old. Exclusion criteria: bone disease, use of steroid medicine, post-traumatic, rheumatoid, psoriatic osteoarthrisis or contractures due to tenosynovitis of the palmar flexors	Only a train set (no validation or test set)	Instances with missing data are removed depending on the AI model.
Karasik et al., 2000 [13]	5 756 living human individuals (2 683 males and 3 073 females) belonging to 9 ethnic groups.	Individuals come mostly from autochthonous populations. Subjects' age ranging from 17 to 96 years old Exclusion criteria: same as in Karasik et al., 1999	Only a train set (no validation or test set)	-
Bocaz-Beneventi et al., 2002 [14]	61 deceased human subjects	Postmortem interval ranging from 7 to 144h.	Train set (n = 51 cases) and validation set (n = 10 cases). No external validation.	-
Constantinou et al., 2015 [15]	953 living human individuals (778 males and 175 females)	Prisoners serving sentences for at least 2 years (sexual or violent offences)	Train and validation sets (10-fold cross validation). Gender imbalance in data.	Missing data is inferred from evidence provided by other data within the model and linked to the missing data
Simmons et al., 2016 [16]	13 bones	6 cranial bones from human beings, 7 bones from animals	Only a training set (no validation or test set)	-

Stern et al., 2016 [17]	240 living human individuals	Caucasian male volunteers aged between 13 and 25 years old	30 subjects in validation set and remaining 210 subjects in train set (8-fold cross validation). Data augmentation up to 1 050 instances. Uniform distribution in age in the training sets (declared by author) but slight imbalance in age in the whole dataset (bell-shape distribution).	-
Yilmaz et al., 2017 [18]	44 human fetuses (24 live and 20 stillborn cases)	-	Train (77% of data) and test (23% of data) sets	Missing data is removed
Ebert et al., 2017 [19]	52 deceased human subjects (28 cases of hemopericardium with 20 males and 8 females, 24 control cases with 14 males and 10 females)	Exclusion criteria: advanced decomposition, thoracic trauma, evidence of blunt or sharp force injury, presence of cardiac medical devices, hemothorax	Train (50 % of data) and validation (50% of data) sets generated 20 times using a different randomization approach. No external validation. Gender imbalance in data.	-
Spampinato et al., 2017 [20]	1 391 living human subjects	Subjects from the Digital Hand Atlas Database System. Subjects' age ≤ 18 years old.	Train and validation sets (5-fold cross validation) No external validation. Data balanced in gender and race. Age imbalance in data.	-
Stern et al., 2017 [21]	103 living human individuals (309 3D MRI images)	Male Caucasian volunteers aged between 13 and 25 years old	Train and validation sets (4- fold cross validation) No external validation. Uniform distribution in age.	-
Zhang et al., 2018 [22]	562 living human individuals	Chinese subjects, with no history of chronic illness, trauma, physical deformity, surgical procedure that might affect stature or sternum dimensions. Subjects' age ranging from 20 to 90 years old.	Train (n = 512 subjects) and test (n = 50 subjects) sets. Data balanced in gender. Age imbalance in data.	-
Canturk et. al., 2018 [23]	10 deceased human subjects (1 female, 9 males)	Subjects without corneal opacity affected by cause of death or prone position, no head or cervical trauma or edema. Subjects' age ranging from 30 to 70 years old.	Train and validation sets (10-fold cross validation) or 9 subjects in the train set and 1 subject for test. Data balanced in age, imbalanced in gender.	-
Heimer et al., 2018 [24]	150 deceased human subjects (75 cases with skull fractures, 75 control cases)	Fractures come from accidents, suicides, unknown manner of death, one criminal offense. Controls come from natural death followed or not by accidents and unknown cause. Exclusion criteria: age < 18 years old, shattered skulls lacking resemblance to physiological anatomy, visible residues from surgical intervention	Train and validation sets (2-fold cross validation with random sampling) repeated 100 times. No external validation. Data imbalanced in gender (male-to-female ratio: 70:30) and manner of death (category size ranges from 1 to 55).	-
Koterova et al., 2018 [25]	941 samples from deceased human subjects	Subjects' age ranging from 19 to 100 years old.	Train and validation sets (5-fold cross validation). No external validation.	-

			Data balanced in gender, imbalanced in race	
Matoba et al., 2018 [26]	111 deceased human subjects	Subjects' age ranging from 18 to 95 years old.<br>Postmortem intervals ranging from 0.3 to 60 days.<br>Exclusion criteria: severely corrupted, unconfirmed lung weight, severe putrefaction, time between PMCT and autopsy is more than 6h, age < 18 years old.	No information about the number or the size of the datasets.<br>Data imbalanced in cause of death.<br>Inconsistency between number of cases and number of males and females (more males and females than cases)	-
Stern et al., 2019 [27]	328 3D hand MRI images or 835 2D X-ray hand images both acquired from living human individuals	3D MRI dataset: male Caucasian volunteers (aged between 18 and 25 years old), 141 subjects ≤ 18 years old, no history of endocrinal, metabolic, genetic or developmental disease.<br>2D X-ray dataset: subjects from the Digital Hand Atlas Database and aged between 10 and 19 years old	Datasets: train and validation for each dataset (4-fold cross validation).<br>No external validation.<br>Uniform distribution in age for the 2D dataset.	-
Andersson et al., 2019 [28]	101 deceased human subjects	Swedish indoor settings. Subjects without presence of insect activity, major traumatic damage, submersion or burn	Train on 93 cases and test on 8 cases	Missing values or values deemed potentially biases are assigned a null value.
Avuclu et al., 2019 [29]	1 315 teeth images from orthopantomograms of human subjects (not clear if they are deceased or alive)	162 different age groups ranging from 4 to 63 years old.	Number of images in train set is not clear. The test set is composed of less than 12 images.	-
De Back et .al, 2019 [30]	> 12 000 orthopantomograms collected from living human individuals	Subjects aged between 5 and 25 years old.	Train (75% of data) and test (25% of data) sets.<br>No external validation but no mention of hyperparameter tuning.	-
Li et al., 2019 [31]	1 875 living human subjects	Participants from West China Han group.<br>Subjects aged between 10 and 25 years old.<br>Exclusion criteria: evident deformities or disease in the pelvic region	1 498 images in the training set and 377 in the test set.<br>Balanced data in gender.<br>Age groups of 1-year intervals. Slight imbalance in age. Images with superposition abdominal organs over the iliac crest removed from train set but kept in test set.<br>No external validation.	-
Milosevic et al., 2019 [32]	4 000 panoramic dental X-ray images from living human individuals	European subjects (Caucasian) aged between 19 and 85 years old.<br>Female-to-male ratio: 58,8/41,2	Train and validation sets (77% of data) and test set (23% of data). 10-fold cross validation for performance evaluation on all the data (train, validation and test sets gathered into a sole set).<br>Data imbalanced in age.	-
Turan et al., 2019 [33]	284 living human individuals	Subjects aged between 24 and 60 years old.<br>Exclusion criteria: operation, subluxation, presence of bone fracture or deformities.	Train set (80% of data) and validation set (20% of data) shuffled at each iterations.<br>No external validation.<br>Data balanced in gender.	Authors declared instances with missing data are not suited for linear discriminant

				analysis
Abderrahmane et al., 2020 [34]	190 living human subjects (11 076 hand photographs)	Subjects aged between 18 years old and 75 years old	Train set (70% of data) and validation set (30% of data).<br>Balanced data in age after balancing (ages are 18 to 26, 28 to 30, 36, 43, 54, 70, 75 years old).<br>Data imbalance in age, gender, skin triplet.<br>About 710 images per age subset.<br>No external validation.	-
Garland et al., 2020 [35]	50 deceased human subjects (25 cases with fatal head injuries, 25 suicides by hanging)	Transport related and accidental fatal deaths for cases with fatal head injuries.<br>Exclusion criteria: suspicious, homicidal and deaths of children (age < 10 years old) due to potential legal issues, signs of decomposition, cases with neurosurgical procedures.	Train and validation sets (40 subjects: 20 cases with fatal head injuries and 20 controls, validation on 20% of the training set) and test set (10 subjects: 5 cases with fatal head injuries and 5 controls).<br>Data imbalanced in gender (male-to-female ratio is 19/6 for cases with fatal head injuries, 22/3 for controls)	-
Homma et al., 2020 [36]	280 deceased human subjects (140 drowning cases corresponding to 3 784 images and 140 non-drowning cases corresponding to 3 863 images)	-	Train and validation sets (10-fold cross validation with balanced data in drowning distinction and same size for each fold)	-
Peleg et al., 2020 [37]	461 deceased human subjects	No individual with more than 2 absent ribs, no measurements from broken or deformed ribs (train set)	Train set (413 subjects, unbalanced in gender, European Americans and African American aged between 20 to 87 years old), leave-one-out cross validation set (33 adults, balanced in gender, aged between 10 and 60 years old, no race given), 15 adults for validation of virtual measures (no race given)	Instances with 1 missing rib are kept since models are usable for individual ribs.
Pena-Solorzano et al., 2020 [38]	450 deceased human subjects	Subjects aged between 20 years old and 90 years old.<br>Inclusion criteria: males only, cause of death as natural disease or drug overdose (to avoid physical trauma cases)	Train set (70% of data), validation set (15% of data), test set (15% of data) with 5 times random subsample for localization of femur and 8 times for feature classification.<br>Imbalance in feature classification.	-
Tirado et al., 2020 [39]	11 living human individuals (4 women and 7 men)	Subjects aged between 22 and 68 years old	Train set (1 712 images), validation set (215 images) and test set (213 images). 10-fold cross validation is used on the train set.<br>Data imbalanced in bruise location and age.<br>Data balanced between validation and test sets but imbalanced for each class (including train set)	Missing data is not handled
Vila-Blanco et al., 2020 [40]	2 289 living human subjects	Spanish Caucasian subjects aged between 4.5 years old and 89.2 years old.	Train and validation sets: 8-fold cross validation with test as isolated fold and train	-

			and validation as the 7 other sets (train: 80% of data, validation: 20% of data). Imbalanced data in age (963 cases for 10-19 years old and 31 cases for 70-89.5 years old). More than 20% females than males.	
Mauer et al., 2021 [41]	79 living human subjects for the coronal dataset, 297 for the sagittal dataset.	Caucasian males with middle to high socio-economic status, raised in Hamburg (Germany) or surroundings, aged between 13 and 21 years old, and no chronic diseases or severe bone<br>Injuries.<br>Coronal dataset: male Caucasian subjects aged between 14.41 and 21.66 years old.<br>Sagittal dataset: male Caucasians subjects aged between 13 and 21.83 years old.	Coronal dataset: 2 220 images split into train (66%), validation (18%) and test sets (19%, stratified 5-fold cross validation).<br>Sagittal dataset: 404 images.<br>Data unbalanced in age (age-stratified data augmentation)	Missing data is removed if bone info < 2%
Ozdemir et al., 2021 [42]	No information about the number of cases (204 radiographs)	Exclusion of 0-7 month old subjects<br>KCRD dataset: individuals from hospitals in Kütahya (Turkey) aged between 0 and 18 years old.<br>RSNA dataset: individuals with mean age of 127.32 months	Datasets split into training, validation and test sets with proportion 0.7/0.15/0.15 respectively.<br>Data unbalanced in age.	-
Oura et al., 2021 [43]	19 piglet carcasses	Piglet weights range from 2.05 to 4.76 kg, piglets died from natural death and are stored 5 days maximum. No overlap of gunshot wounds.<br>Exclusion criteria: external deformability and abnormal or blotchy skin pigmentation	Dataset is composed of 60 negative controls, 50 contact shots, 49 close-range shots and 45 distant shots images.<br>Dataset split into training, validation and test sets with proportion 0.6/0.2/0.2 respectively.<br>No external validation.	-
Garland et al., 2021 [44]	Number of cases is not provided.	Exclusion criteria: autolysis, marked decomposition, postmortem bacterial overgrowth, processing artifacts, fading of stains, early acute myocardial infarction (1 day old), healing myocardial infarction and other causes of myocardial scarring	50 images of normal heart slides, 50 images of old myocardial infarction slides and 50 images of acute myocardial infarction slides.<br>Train, validation and test sets with 108, 15 and 30 images respectively.<br>No label imbalance.<br>No external validation.	-
Ibanez et al., 2022 [45]	195 postmortem cases	55 females with median age of 64 years old and 140 males with median age of 54 years old.<br>Exclusion criteria: signs of advanced decomposition, organ explantation, severe trauma with extensive damage to the corpse such as amputation or exenteration, deviating scanning protocol, no PMCT data, rib fracture present in the volumetric CT data and the autopsy but not visible in the rib unfolding tool or in which the rib defect was in the cartilaginous part of the rib, still under investigation	5-times split into 2 sets (train/validation and test sets) then 5-fold stratified cross validation on the train/validation set (85% of all data) split into 344/86 images then test on 77 images (15% of all data)	-

Li et al., 2022 [46]	1226 males and 896 females	Chinese Han individuals from the West China Han group without showing any deformity or diseases in the femur region, and aged between 18 and 26 years old.<br>Caucasian population.	Train/validation (1915 images) and test (207 images) sets.<br>2 test datasets: 361 pelvic radiographs from Han pop (207 individuals aged from 18 to 26 years old 154 individuals aged from 27 to 80 years old) and 50 pelvic radiographs from Caucasian pop (23 individuals aged from 18 to 26 years old and 27 individuals aged from 27 to 80 years old).<br>Data unbalanced in age.	-
